# Assessing Greenhouse Gas Emissions and Health Co-Benefits: A Structured Review of Lifestyle-Related Climate Change Mitigation Strategies

**DOI:** 10.3390/ijerph14050468

**Published:** 2017-04-27

**Authors:** Vivian G. M. Quam, Joacim Rocklöv, Mikkel B. M. Quam, Rebekah A. I. Lucas

**Affiliations:** 1 Department of Public Health and Clinical Medicine, Epidemiology and Global Health, Umeå University, 901 87 Umeå, Sweden; vivianmcw@gmail.com (V.G.M.Q.); joacim.rocklov@umu.se (J.R.); mikkel.quam@umu.se (M.B.M.Q.); 2 College of Veterinary Medicine, University of Georgia, Athens, GA 30602, USA; 3 School of Sport, Exercise and Rehabilitation Sciences, University of Birmingham, Birmingham B15 2TT, UK

**Keywords:** diet, active transport, co-benefits, climate change mitigation

## Abstract

This is the first structured review to identify and summarize research on lifestyle choices that improve health and have the greatest potential to mitigate climate change. Two literature searches were conducted on: (1) active transport health co-benefits, and (2) dietary health co-benefits. Articles needed to quantify both greenhouse gas emissions and health or nutrition outcomes resulting from active transport or diet changes. A data extraction tool (PRISMA) was created for article selection and evaluation. A rubric was devised to assess the biases, limitations and uncertainties of included articles. For active transport 790 articles were retrieved, nine meeting the inclusion criteria. For diet 2524 articles were retrieved, 23 meeting the inclusion criteria. A total of 31 articles were reviewed and assessed using the rubric, as one article met the inclusion criteria for both active transport and diet co-benefits. Methods used to estimate the effect of diet or active transport modification vary greatly precluding meta-analysis. The scale of impact on health and greenhouse gas emissions (GHGE) outcomes depends predominately on the aggressiveness of the diet or active transport scenario modelled, versus the modelling technique. Effective mitigation policies, infrastructure that supports active transport and low GHGE food delivery, plus community engagement are integral in achieving optimal health and GHGE outcomes. Variation in culture, nutritional and health status, plus geographic density will determine which mitigation scenario(s) best suit individual communities.

## 1. Introduction

Climate change and non-communicable diseases are global crises that threaten life as we know it and require a global response. The United Nations’ sustainable development goals (SDGs) highlight the need for a multifaceted, coordinated effort to limit global temperature rise to 1.5–2 degrees Celsius and prevent the catastrophic effects of climate change [1]. Among the stated objectives for health, the SDGs include increasing control and comprehensive treatment of high-burden non-communicable disease through both individual healthy life choices and public policies. The report specifically identifies the need for improved nutrition, healthy cities, and lower pollution, among other factors, in order to obtain health goals, including combating the ever increasing global obesity epidemic [1]. Importantly, there are opportunities to improve both greenhouse gas emissions (GHGE) and health outcomes through climate change mitigation action. This collateral beneficial relationship is termed “co-benefits”. To date, the health co-benefits of several climate change mitigation strategies have been explored extensively. Much attention has been given to large-scale projects pursued at a country or municipal level, such as adoption of cleaner burning fuels, increased resources for reproductive health, and building sustainable infrastructure [2]. However, relatively little attention has been paid to lifestyle co-benefits choices made at the individual level. As individuals and communities strive to design and participate in healthier more sustainable lifestyles, increased research scrutiny is needed to inform decision making lifestyle-related mitigation strategies, which for the purpose of this article, include increased active transport (i.e., cycling and walking) and climate-conscious diet modification (i.e., consuming food stuffs with relatively low GHGE). 

The impact of lifestyle choices on GHGE should not be underestimated or ignored. Currently, carbon-based transportation and some food’s production and transportation significantly contribute to regional and global GHGE. For example in the USA, 9% of GHGE have been attributed to the agriculture sector and 27% to the transportation sector [3]; whereas at a global level, 24% of total GHGE have been attributed to agriculture, forestry and other land use and 14% GHGE to transportation [4]. Such figures highlight how lifestyle-related mitigation strategies can significantly reduce GHGE. Moreover, lifestyle-related mitigation also provides unique insight into the challenges of measuring and altering individual behavior and decision-making. As a result, lifestyle-related mitigation strategies need to be tailored to affect individual decision-making as well as policy and community development. This requires accurate, relevant and meaningful information for maximal societal engagement [5]. 

Public engagement must also be fostered with respect to physical activity, diet and health. It is estimated that a third of adults and four-fifths of adolescents do not meet current public heath recommendations for physical activity [6]. In addition to this, there is also a global nutrition transition where traditional plant-based diets rich in fruit and vegetables are being replaced with diets that are rich in calories provided by animal fats and sugar and are low in complex carbohydrates [7]. Subsequently, unhealthy diets and physical inactivity are among the leading causes of the major non-communicable diseases and contribute substantially to the global burden of disease, death and disability [8]. Yet, physical and dietary interventions have the potential to prevent, slow the progression or even treat the majority of non-communicative diseases. Thus, there is major potential for improving public health and well-being through healthier diets and physical activity.

Increased physical activity, decreased sedentary time and healthy dietary choices are modifiable behaviors that can reduce morbidity and premature mortality as well as decrease GHGE when the necessary policies and infrastructure exist for sustainable choices. However, to date, lifestyle-related mitigation co-benefits appear to have received less consideration than other co-benefits strategies. Indeed, the IPCC’s recent Summary for Policy Makers lacks any discussion of active transport and diet co-benefits review articles [2]. Though model based diet [9] and active transport [10] co-benefits studies have separately been reviewed, to date both sets of literature have not been collectively examined. Given the interconnectedness of diet and exercise and their combined influence on health and the global burden of disease, an examination of the current literature with a particular focus on health outcome analysis is needed. Broad-scale implementation of policies to encourage these sustainable lifestyles, require a thorough review of the literature to identify the most effective strategies for obtaining health and climate change mitigation outcomes. Therefore, the aim of this review was to identify and summarize the existing research on lifestyle choices with the greatest potential to mitigate climate change while improving health. The information gained could aid individual decision-making, as well as the development of health, transportation, and environmental policy.

## 2. Methods

### 2.1. Selection of Articles for Review

#### 2.1.1. Literature Search

Two separate searches were conducted, one for active transport health co-benefits literature and one for dietary health co-benefits literature. In both cases, searches were performed in ScienceDirect (1995 to present), PubMed (1940s to present), and Google Scholar. The last search was performed 7 July 2014. For the active transport literature, search terms included greenhouse gas, emissions, health, co-benefits, active transport, and active travel. The primary search in ScienceDirect was as follows: (“greenhouse gas” OR emissions) AND health AND co-benefit AND (“active transport” or “active travel”). For the dietary literature, search terms included greenhouse gas, emissions, health, co-benefits, food, and diet. The primary search in ScienceDirect was as follows: (“greenhouse gas” OR emissions) AND food AND diet AND health AND (“co-benefit” or cobenefit). Articles that were deemed irrelevant based on title and abstract were excluded. The full text of the remaining, relevant articles was retrieved and further reviewed for inclusion based on the pre-defined inclusion criteria described below. Relevant articles cited within the literature were also sought out and analyzed for inclusion. 

#### 2.1.2. Inclusion Criteria

Articles on greenhouse gas mitigation strategies related to lifestyle choices with health co-benefits were of interest. The lifestyle-related mitigation strategies in the existing literature include active transport and diet. For inclusion, an article must have quantified both the GHGE and the health or nutrition impact of at least one of the lifestyle-related mitigation strategies. The most common reason for exclusion was quantification of either (as oppose to both) the GHGE or the health or nutrition impact of at least one of the lifestyle-related mitigation strategies. English language articles published in peer-reviewed journals were considered without date restrictions. Review articles were not included. Due to the wide range of GHGE and health outcome measurements of interest, meta-analysis was not a goal of this review. 

### 2.2. Data Extraction

We created a data extraction tool guided by the PRISMA guidelines. Two authors tested the tool on a subset of the literature and revisions were made until the tool design was agreed upon. The evaluation included: authors; publication date; study population description, including age, gender, time and geography; baseline data sources; study design; health and GHGE analysis methods; and outcomes. 

Two authors performed a second evaluation of each article using a rubric. This rubric assigned a score based on the article’s success in recognizing and addressing the biases, limitations and uncertainties of the research according to three different categories: (1) general; (2) GHGE; and (3) health (see scoring criteria in Table 1). Articles received a binary score for meeting or failing to meet each of the criteria and were evaluated by two of the authors independently. Following evaluation of all selected articles, rubric outcomes for each article were compared and any discrepancies between the two authors’ scoring were resolved by a previously agreed upon arbitrator if necessary. Accordingly, articles were assigned a final score in each of the three categories (general and GHGE categories were scored from zero to five and the health category was scored from zero to ten). The rubric was derived from that used by Yip et al. [9] and adapted to apply to a wide range of active transport and dietary articles. 

## 3. Results

### 3.1. Article Retrieval

**Figure 1:**
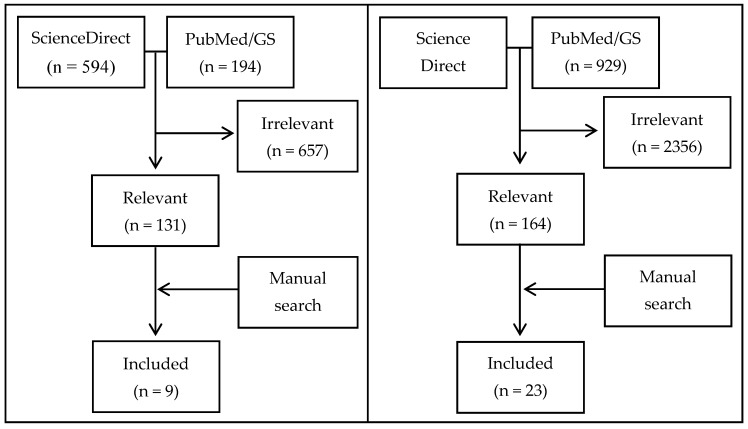
Identification and inclusion of relevant articles; GS = Google Scholar.

Figure 1 shows the identification and inclusion of articles selected for this review.

#### 3.1.1. Active Transport Article Retrieval

The majority of active transport articles retrieved from the ScienceDirect search did not address greenhouse gas mitigation strategies related to active transport. For relevant articles, the full text was retrieved. Most were review articles or only examined one of the two outcomes of interest (i.e., active transport impacts on health or greenhouse gas emissions impacts on health, but not both). Five articles from the ScienceDirect search meet the inclusion criteria. Subsequent searches on PubMEd and Google Scholar returned two additional articles. A manual search based on articles cited in the selected literature added a further two articles. In total, literature searches produced nine articles, eight of which specifically addressed active transport, and one of which addressed both active transport and diet.

Of the nine selected active transport articles, two each were based on UK, U.S., and New Zealand data, and one each on Indian, Spanish, and Organization for Economic Co-operation and Development (OECD) country data. For health outcome measures, two articles estimated the disability-adjusted life years (DALYs), another two articles evaluated monetized gains from reduced mortality or increased life expectancy, and four articles estimated all-cause mortality with or without evaluation of specific disease occurrences. One article correlated increased trends in sedentary behavior and meat consumption with obesity [11]. Identified health determinants of morbidity and mortality outcomes included increased activity, exposure to pollutants and traffic injuries. Most articles utilized the World health organization’s HEAT tool to estimate Metabolic Equivalents of Tasks (METs) associated with active transport. In addition to evaluating the health impact of increased activity, most articles also considered exposure to pollutants and traffic accidents under different transport scenarios. All included articles estimated GHGE using measured carbon dioxide (CO_2_) equivalent emissions based on regional, country, or municipal models as applicable to the population of interest.

#### 3.1.2. Diet Article Retrieval

The majority of diet articles returned from the ScienceDirect search did not address greenhouse gas mitigation strategies related to diet. For relevant articles, the full text was retrieved. Most were review articles or only examined one of the two outcomes of interest (i.e., either diet change impacts on health or greenhouse gas emissions impacts on health, but not both). Eight articles from the ScienceDirect search were selected for inclusion. Subsequent searches on PubMed and Google Scholar resulted in ten additional articles for inclusion. A manual search based on articles cited in the selected literature added a further four articles. The Michaelowa and Dransfeld [11] article retrieved during the active transport search was also included as it detailed diet changes too. Therefore, literature searches produced a total of 23 diet articles.

Of the 23 selected diet articles, seven were based on UK data, three were based on Swedish data, two each were based on Brazilian, French, Danish, and European data, and one each on New Zealand, Italian, Dutch, OCED, and Australian data. For health outcome measures, 18 articles evaluated intermediate determinates of health including consumption of cholesterol or saturated fats, nutrients and total calories. Several articles went further to estimate health outcomes directly. Five estimated changes in mortality or DALYs, and three evaluated the occurrence of specific diseases. All articles estimated changes to CO_2_ equivalents or GHGE. Other environmental outcomes, such as change in land use, were also occasionally included.

Diet articles included, with the exception of two, detailed estimates of their proposed GHGE reductions under various scenarios and therefore quantified reductions could be reported (Table 2). Masset et al. [31] did not model scenarios or estimate a specific level of GHGE reduction, but compared estimates of GHGE for commonly consumed foods based on reported dietary intake (i.e., each food item was assigned an GHGE value (expressed in #gCO_2_eq/100 g) that was compared to the nutritional value of the item to obtain a sustainability score). González et al. [26] similarly did not report estimates for modeled scenarios, but rather compared the GHGE and nutritional value of different food items. Between articles, the scale of GHGE reduction varied with geography, population size and the scale of scenarios included. Variation in units and methods precluded any direct comparison between articles.

### 3.2. Active Transport—Summary of Findings

All active transport articles considered scenarios where biking, walking or both replaced vehicular transport. In addition to active transport mitigation strategies, four articles included low emission vehicles in their transportation scenarios and two considered increased public transportation (Table 3). A further two articles concluded that public transport would increase in their proposed healthier and lower emission transport scenarios, but did not calculate the impact. Evaluated health determinants for active transport articles are presented in Table 3. All active transport articles included physical activity as a health determinant in their analysis, whereas traffic injuries were less frequently evaluated (in 7/9 articles). All but one article considered air pollution as a health determinant. Supplementary File, Table S1 details measured outcomes for all included articles.

### 3.3. Diet—Summary of Findings

In selected diet articles, the primary method for reducing GHGE and improving health outcomes was reduced consumption of meat and other animal products. Eleven of the articles only considered meat and other animal product reduction, while twelve considered meat and other animal product reduction amongst additional diet modifications. Further detailed information on each article is summarized in the Supplementary File, Table S1. The frequency with which meat reduction scenarios were considered in selected diet articles is presented in Supplementary File, Table S2. All but four articles considered substitution of meat consumption with alternative food sources. See Table 4 for details on meat reduction and substitution approaches.

### 3.4. Analysis of Biases, Limitations, and Uncertainties—Rubric Scores

**Figure 2:**
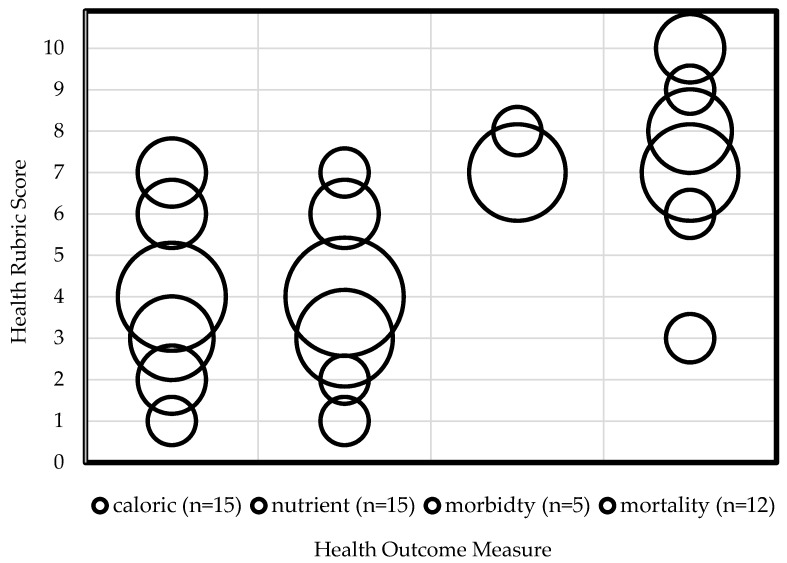
Aggregated health rubric scores by health outcome measure. The area of each circle denotes the number of articles represented at each rubric score (vertical axis), within the health outcome (horizontal axis). Note: As some articles included multiple measures (i.e., mortality and morbidity), their health scores are reported in all relevant categories.

Lifestyle-related rubric scores (Table 1) are summarized in Table 5. Diet and active transport articles scored better in the GHGE category than in the health category of the rubric, although this difference was negligible for active transport. These scores indicate that the GHGE effects of lifestyle-related mitigation strategies are more consistently measured without biases, limitations or uncertainties than are the health effects. Additionally, the health effects measured in the diet articles had a lower rubric score than the health effects measured in the active transport articles. In addition to discrepancies in health scores, the diet and active transport articles also differ in health outcome measures as reported in Figure 2 and Table 6. 

#### 3.4.1. Health-Related Biases, Limitations, and Uncertainties Rubric Scores

Figure 2 aggregates each article’s score from the health category according to the health outcome measured (caloric, nutrient, morbidity or mortality), and Table 6 shows the average health score and range of health scores for each health outcome. Morbidity was the least commonly used health outcome measure, while caloric, nutrient, and mortality outcomes were all used with similar frequency. Articles reporting mortality and morbidity had a higher health score than those reporting caloric and nutrient outcomes. Active transport articles only reported mortality and morbidity health outcomes. Diet articles reported some morbidity and mortality, but primarily reported caloric and nutrient health outcomes. Since some articles included multiple measures (i.e., mortality and morbidity), their health scores are reported in all relevant categories. For a summary of health outcomes included for each article, see Supplementary Table S3. 

#### 3.4.2. GHGE-Related Biases, Limitations, and Uncertainties Rubric Scores

For active transport articles, the average GHGE rubric scores for increased public transport articles (*n* = 2) was 3.5/5. For Low Emissions Vehicles articles (*n* = 4) it was 3.25/5 and for active transport only articles (*n* = 4) it was 4/5. Articles that only considered active transport had better GHGE scores as GHGE were reduced more drastically than in scenarios where public transportation or low emissions vehicles were allowed to increase. For diet articles, the average GHGE rubric scores for articles where meat and animal product reduction was only considered (*n* = 2) was 3.9/5, whereas articles where meat and animal product reduction was considered in conjunction with more general diet modifications (*n* = 12) it was 4.5/5. Diet articles that considered more than reducing consumption of meat and animal products had better GHGE scores as other high GHGE producing food groups were also reduced.

## 4. Discussion

This is the first review to identify and summarize research on lifestyle choices that improve health and have the greatest potential to mitigate climate change. A meta-analysis comparing the co-benefits of lifestyle-related climate change mitigation strategies was not feasible due to: (1) the small number of articles found that quantified both GHGE and health impacts associated with lifestyle-related mitigation strategies; (2) the scale and range of lifestyle-related mitigation strategies examined within the literature, plus; (3) differences in model assumptions. However, following a structured review of the literature it seems that the scale of impact on health and GHGE outcomes depends predominately on the aggressiveness of the diet or active transport scenario modelled, versus the modelling technique. Implications for both researchers and policymakers are discussed below.

### 4.1. Analysis of Active Transport Article Findings

All reviewed active transport articles concluded that their scenarios would reduce GHGE and increase physical activity resulting in net positive health outcomes. The degree to which GHGE were reduced or health was improved depended largely on the scale of the transportation modification, the range of health outcomes considered, and the assumptions of the model. In all reviewed articles physical activity was the largest determinant of health outcomes and was always positive. However, assumptions regarding the effects of traffic injury and air pollution varied between articles, resulting in a range of positive and negative health outcome predictions. Woodcock et al. [41] found that traffic injuries (modeled using risk (i.e., cycling injury data), distance and vehicle speed assumptions) decreased in all scenarios. Conversely, Macmillan et al. [14] found that serious injuries and fatalities increase for cyclists (modeled using cycling injury data with a safety-in-numbers effect), causing a net increase in traffic fatalities despite decreased fatalities for car occupants. Such outcomes depend greatly on the assumptions made concerning the effect of increased cycling on the fatality and injury rate of cyclists. Countries where cycling is common, like the Netherlands, have much lower fatality rates than countries with little cycling, like the United States. It is not clear how or when fatality rates decrease with increased rates of cycling, though cycle friendly infrastructure and a “safety in numbers” effect almost certainly contributes. The net health benefit from reduced air pollution was positive for all articles, though Rojas-Reuda et al. [17] and Rabl and De Nazelle [16] identified that not all individuals will be affected equally. In these articles, individuals engaging in active transport had increased fatalities from exposure to air pollutants, while the net effect on the population health was favorable. However, recent findings indicate that the health benefits from physical activity outweigh any ill effects of pollution for individuals engaging in physical activity in urban areas [42]. Notably, some transportation scenarios elected to combine elements of “greener” transportation (including cleaner operating vehicles, increased public transportation and active transport) for the greatest effect (Table 3). This combined approach may provide more realistic transportation alternatives for a larger portion of the population.

### 4.2. Analysis of Diet Article Findings

The extent of emission reduction/increase for a diet scenario depended on the scale of the scenario and assumptions of the model. For example, the scenario used by Aston et al. [19] doubled the number of vegetarians and had the remaining UK population reduce their meat consumption to volumes equivalent to the lowest quintet of current British meat eaters. Berners-Lee et al. [20] were even more ambitious and modeled a total switch to vegetarianism and veganism in the UK. Thus, their estimated GHGE reductions was 27.8 Mt/year compared to 40 Mt/year, respectively. Reviewed diets that lowered GHGE were not universally better for health. Similarly, healthy diets were not necessarily low in GHGE. The linear programming models, as in Wilson et al. [40] and Macdiarmid et al. [30], were developed to select the diet that would optimize health and GHGE outcomes at the same time. However, not all diets generated via this method were considered feasible. Several constraints were needed to develop consumer friendly diets with these models, and such considerations resulted in reduced benefits. For example, a diet that met all nutritional requirements while reducing GHGE by 90% contained only seven foods, all in unrealistic quantities [30]. When constraints were added, such as minimum intakes for fruits and vegetables, 52 different food groups were included, and GHGE were only reduced by 36% [30].

In some articles, diet scenarios were also selected for affordability [40]. Cost consideration was highly valuable in identifying feasible diet alterations. Related to this, reductions in over consumption [40], elimination of food waste [29] and reducing “non-core” foods [28] were also examined in some articles. These strategies are cost effective and reduce GHGE, though only reducing overconsumption had direct health co-benefits. Two articles proposed tax scenarios to monetize the GHGE externalities associated with food production and showed this would reduce GHGE and have health co-benefits [22,24].

Overall, achieving mitigation and health benefits from diet modifications requires an extensive understanding of diet and health interactions, cultural dietary traditions, plus GHGE for culturally/geographically relevant foods. Existing GHGE estimates for food items and production systems could be considered relatively narrow. Further database development of GHGE for food items and production systems would guide the development of sustainable diets (defined as: “…diets with low environmental impacts which contribute to food and nutrition security and to healthy life for present and future generations”) [43]. It is essential that future diet-related co-benefits research focuses on sustainable diets as such diets are protective and respectful of biodiversity and ecosystems; culturally acceptable; accessible; economically fair and affordable; nutritionally adequate; safe and healthy; plus optimize natural and human resources [43]. If individuals and communities are to participate in diet-modifications it is imperative that a reasonable and cohesive diet recommendation is made. Such recommendations should clearly fit the definition of a sustainable diet. For the most part, dietary modifications in the reviewed articles were limited to alternations in meat consumption. Meat production is associated with high GHGE, and many people in OECD countries consume more meat and protein than dietary guidelines recommend. However, more comprehensive diet assessments and modifications can lead to sustainable diets with greater mitigation and health improvements if meat is substituted with the lowest emitting as well as the most nutritious and affordable foods. 

### 4.3. Analysis of Biases, Limitations, and Uncertainties

The reviewed articles also contained parametric and structural uncertainties [44]. Parametric uncertainties arose from: model assumptions concerning the level of diet or active transport modification obtainable, air pollution dispersal model parameters, and relative risk for morbidity and mortality associated with physical activity or diet. Structural uncertainties of the model resulted from assumptions about exposure and health outcome relationship (linear vs. non-linear), identification of relevant health outcomes, safety-in-numbers effect on cycling injury and fatality, and inclusion of the appropriate food items and categories. Most articles included sensitivity analysis methods such as stochastic Monte Carlo analysis or inclusion of multiple model formulations to address some of these uncertainties. It has been asserted that there are four primary sources of uncertainties in modeling health co-benefits: (1) simulating change in health determining exposures over space and time; (2) following change in exposure, determining the time response for health effects; (3) comparing mitigation strategies and their health effects over time within a population; and (4) establishing burden of disease projections into the future in the absence of mitigation [44]. These sources of uncertainty were demonstrated in the reviewed articles. 

In the simulation of change in a health determining exposure (point 1 above), the feasibility of widespread and extensive diet or transportation modifications over space and time must be addressed and was in some articles. Linear programming models incorporated constraints to improve the acceptability of their proposed diets, whereas, Aston et al. [19] and Berners-Lee et al. [20] suggested levels of vegetarianism and meat reduction that seem overly optimistic. It is imperative that research focuses on sustainable diets that are applicable for the geographic region of interest and practical to present to policymakers and stakeholders. Similarly, commuting distances make it unlikely that US cities will reach the levels of active transport seen in the Netherlands, unless city densities increase significantly. The challenge weather poses to increasing physical activity in certain locations and seasons should also be considered, particularly in the context of climate change [45]. 

A change in activity level or diet (the “exposure” referred to in point 2 above) will not have an immediate effect on health, and effects may change over time. Unintended outcomes must be considered when determining changes in health or GHGE following a diet or transportation modification. Macmillian et al. [14] included a system dynamics model to assess many of these potential feedback mechanisms: safety in numbers resulting from increased cyclists; change in vehicles speed due to reduced numbers; and perceived and actual risk of injury following transportation mode change and safety measures. It was generally assumed that accommodating infrastructure, such as cycle lanes, would lead to an increase in active transport. However, participation in active transport may replace other physical activities, leading to neutral or reduced net activity levels (e.g., a longer active transport commute may reduce time and energy for regular exercise regimes). Conversely, there was evidence that active transport was associated with generally higher physical activity levels, though the causative link in such an association is yet to be established [46]. With diet modification, reduction in meat consumption may lead to poor health outcomes if unhealthy foods replace meat in the diet. Well-designed and effective interventions require thorough analysis of these feedback mechanisms and system dynamics modeling could be a very useful tool. 

As per point 3, diet and physical activity changes may affect individuals within the population differently depending on age, sex, and baseline activity and diet. Additionally, food production, automotive technology, and medical practices are continually developing, thus estimates of the impact of diet modification and active transport will change over time. The GHGE estimates produced in these articles may become less reliable as technology advances. It is also important to recognize that population-based health outcomes could improve with better medical intervention, independent of exercise or diet modification. This potential impact on the level of disease burden within a population is a source of uncertainty (point 4). Estimations of the future burden of disease were not attempted in these models, though some models incorporated this uncertainty in their future projections according to published burden of disease estimates [12,13,14,15,16,17,18,19,21,22,25,34,41]. 

Despite the prevalence of uncertainties, the multi-potent disease prevention and treatment potential of diet and physical activity interventions should not be overlooked. Indeed, diet and physical activity/exercise interventions would make for a healthier, more resilient general population, with the likely flow-on benefit of reduce hospital admissions and reduced GHGE from the health sector. Thus, diet modifications and active transport will continue to improve GHGE and health outcomes, however, the scale of their effect will change over time and individuals will not be impacted equally by these mitigation strategies.

#### 4.3.1. GHGE

On average, diet and active transport articles scored better on the GHGE section than on the health section of the biases, limitations, and uncertainties rubric, although this difference was negligible for active transport (Table 5). This is likely because quantifying diet and exercise health outcomes is more challenging than quantifying changes in GHGE. Each reviewed article produced a GHGE estimate based, at least in part, on CO_2_ equivalent emissions. Conversely, health outcomes in the dietary articles ranged from morbidity and mortality to intermediate determinants of health such as nutrient and calorie intake. In the majority of diet articles, health outcomes were associated with nutrient or calorie intake, whereby improved nutrient profiles or reduced calorie intake was associated with improved health outcomes; however, the exact impact of the diet modification on morbidity and mortality was not quantified. A few articles use previously published relative risks related to specific nutrient intake to estimate morbidity and mortality associated with proposed diet changes (Table S3; [12,13,14,15,16,17,18,21,22,25,34,41]. Alternatively, active transport articles only used mortality and morbidity health outcomes. Subsequently, the uncertainty surrounding dietary health outcomes was greater than that for active transport and therefore active transport articles scored better in the health section of the rubric (Table 6). 

The health category was heavily weighted in the scoring rubric due to the focus of the current review and this may have contributed to the lower rubric scores assigned to the health section. Nonetheless, it would seem that measuring health outcomes with fewer biases, limitations, and uncertainties is an area where improvement is needed, particularly when modelling diet co-benefits. Focusing on techniques to measure morbidity and mortality would strengthen health analyses for diet co-benefits and provide standard measures that could be compared across articles. 

#### 4.3.2. Active Transport

Transportation-related emissions are a significant contributor to global GHGE. In this review, “greener” transportation options estimated GHGE as almost zero for active transport, a lower per capital emissions level for public transportation, or as a lower greenhouse gases emit per kilometer traveled for cleaner operating vehicles. To estimate transportation-related emissions in a given geographical area, traffic patterns and air dispersal patterns were estimated via generalized tools such as the SIM-air Model developed by UrbanEmisssions.info or the COPERT4 software from the European Environment Agency.

Active transport articles did not used lifecycle analysis techniques to quantify GHGE. Instead, GHGE were based off levels of vehicular transportation and the resultant release of CO_2_ and other pollutants. Such GHGE estimates can be considered relatively straightforward as tail-pipe emissions are considered the primary source of emissions in vehicular travel and methods for estimating them are generally agreed upon. Therefore, active transport articles were able to focus on this aspect alone, without considering the lifecycle analysis of producing and maintaining vehicles for transport [16]. 

Active transport articles all estimated morbidity or mortality associated with physical activity, but estimations varied as to whether they also include the effects of pollutant exposure and traffic accidents on heath (Table 3). The impact of physical activity on health outcomes was estimated using the HEAT model, comparative risk assessment, or systems dynamics modeling (Table S4). Many of the active transport articles incorporated PM_2.5_ or PM_10_ (particulate matter with aerodynamic diameter of 2.5 μm or less or 10 μm or less, respectively) into their analysis of health outcomes as a measure of pollutant exposure. Although it is not the only pollutant with a health impact, PM is considered the most relevant factor in predicting health outcomes [47]. Exposure to pollutants for the community at large was considered in all but one article, Michaelowa and Dransfeld [11]. Many models also estimated changes in traffic accidents under various scenarios and their impact on health. Grabow et al. [12] and Michaelowa and Dransfeld [11] were the only articles that did not consider traffic injuries.

#### 4.3.3. Diet

The majority of reviewed diet articles estimated GHGE using lifecycle assessments (LCAs) of food items or groups. Some articles used a top-down methodology with input-output analysis to scale self-reported consumption data to match food supply [20,32]. This top-down and bottom-up hybrid model accounted for food waste and underreporting of consumption, giving a more accurate GHGE profile of the food supply. In diet articles, the primary method for reducing GHGE and improving health outcomes was to reduce consumption of meat and animal products. Limiting diet modifications to reduced meat and animal products is a practical first step because the health and GHGE impact of meat, particularly beef and processed meat, is significant and widely agreed upon. However, if meat consumption is reduced practical replacements need to be identified as individuals are likely to look for alternative calorie sources. Many of the articles considered substituting meat with produce and grains. This is a much more challenging task, as performing LCAs is laborious. Knowing which foods and production systems produce lower GHGE is essential for making recommendations for a sustainable diet. Also, while many people in OECD countries consume an excess amount of meat and protein, excess consumption is not ubiquitous. It is important that appropriate alternatives for beneficial meat nutrients (i.e., protein, iron, B vitamins) are identified in substitute diets in order to make sustainable diet recommendations. The co-benefits of reduced meat and consequently reduced calorie intake are evident but different from that of an isocaloric alternative diet.

Quantifying GHGE across stages of food production and delivery (i.e., from the farm, to processing, to consumers) can be complex. Each stage provides a variable, but significant contribution to GHGE. Many of the GHGE estimates in the included articles relied on previously published databases of food group’s GHGE estimates, with varying degrees of generalization regarding the source and delivery point of food items. The challenging nature of tracking the GHGE impact for individual food items likely contributes to the focus on meat production in the included dietary articles. Meat is known to be a significant contributor to agricultural and food-related GHGE and is a significant source of dietary calories, protein, and fat. It has been estimated that up to 35% of GHGE come from agriculture and land use for livestock production [48]. 

#### 4.3.4. Methodological Issues

The article selection may be biased as articles included were all published in English language, peer-reviewed journals. Our analysis of included articles relied heavily on the rubric of biases, uncertainties, limitations described in Table 1. The rubric was generalized to apply both to diet and active transport articles and may not identify every area of importance in evaluating each body of literature. Also, by limiting our review to articles that examine both the health and GHGE outcomes, contributions from articles that examine only one of the two outcomes were excluded. 

## 5. Conclusions

This review aimed to identify opportunities for health benefits and GHGE reductions through lifestyle modification. It also highlighted similarities in the two lifestyle-related mitigation strategies. The novel aspects of this review are: (1) the collective focus on both diet and transport co-benefits (2) the evaluation of similarities and differences in these bodies of literature, plus (3) the assessment of biases, limitations and uncertainties of included articles.

### 5.1. Implications for Research

Modeled scenarios of increased active transport conclude that health and GHGE outcomes can be improved. However, the best methods for bringing about transportation change are less clear. Macmillan et al. [14] used systems dynamic modeling to examine the dynamic effects of proposed transport policies. Their model incorporated inputs from community, academic, and policy stakeholders and therefore, seems to have considered the most feasible transport modification possible for the assessed region. Participation between interested parties is needed to identify the best interventions for diet and active transport.

For dietary interventions, the outcomes for health and GHGE are less certain. A more exhaustive list of LCAs for food items is needed in order to identify and promote low-GHGE meats and meat substitutes that best meet nutritional requirements. As far as achieving diet modification, the acceptability of taxation on high GHG emitting food will vary greatly between countries. In some countries this may provide an ideal mechanism for achieving a sustainable diet, though other strategies such as limiting meat production or placing restrictions on fats and sugars included in processed foods also have potential. Health outcome estimates could be improved with greater understanding of the effect of diet on morbidity and mortality.

### 5.2. Guidelines, Advice for Policy Makers

It is difficult to draw clear conclusions from the current body of literature regarding the scale of GHGE reductions and health improvements from potential diet and active transport interventions. Outcomes presented in the reviewed articles are difficult to compare directly due to variations in the assumptions and measurement units. More attention needs to be given to developing realistic and relevant diet and transportation modifications strategies. To date, the most effective approach proposed in the literature is the combination of a Pigouvian tax for GHGE and a “sin” tax on unhealthy food items. A tax on GHGE alone will not necessarily result in a healthier diet. Meat consumption reduction strategies may be best suited to countries with excess consumption of meat and protein, while others may need to consider developing low GHGE meat production systems that can supply their nation with a healthy protein source.

## Figures and Tables

**Table 1:** Rubric for scoring biases, limitations, and uncertainties.

**Criteria for the General Category**	
1.	Acknowledges uncertainty in the feasibility of dietary or transportation shift within the population
2.	Acknowledges uncertainties in factors and unit measures used in the analysis
3.	Recognizes uncertainties in factors and values used in the analysis are subject to change over time
4.	Acknowledges uncertainties of outcomes
5.	Uncertainties identified were subject to appropriate sensitivity analysis
**Criteria for the Emissions Category**	
1.	Emissions factors account for most life cycle stages
2.	Emissions factors included are appropriate and include more than carbon dioxide
3.	Acknowledges modeling errors for emissions factors
4.	Emissions factors and indicators are selected for the appropriate geographic location
5.	Acknowledges possibility of unintended emissions outcomes
**Criteria for the Health Category**	
1.	Acknowledges uncertainties in baseline data
2.	Acknowledges uncertainties in modeled relationship between exposures and health outcomes
3.	Acknowledges uncertainties and complexities in nutrient/physical activity’s impact on health outcomes
4.	Acknowledges possibility for other, unintended health outcomes
5.	Indication of generalizability of the health outcome in terms of the population demographic characteristics
6.	Indication of generalizability of the health outcome in terms of population dietary/transport characteristics
7.	Acknowledges the possibility of residual confounding associated with the meta-analysis derived parameters
8.	Indicated uncertainty concerning the time needed for health effects to be observed
9.	Indicated possibility of double counting or over estimating effects
10.	Indicated that dietary/transport shifts will affect the health of some groups more than others

**Table 2:** Included articles, inclusion criteria, and emissions reduction estimates.

Authors, [Reference], Year, Location	Emissions Outcome	Emissions Reduction in CO_2_ eq (% Reduction)	Health-Linked Outcome
Lifestyle-Related Mitigation Strategy—ACTIVE TRANSPORT			
Grabow et al. [12], 2012 Midwest US	O_3_, PM_2.5_	1.8 tetragrams/year (20% reduction in vehicle emissions)	Morbidity, Mortality
Lindsay et al. [13], 2011 NZ	CO_2_ eq	8695 tonnes/year (30% shift of short-distance car trips to bicycle)	Morbidity, Mortality
Macmillan et al. [14], NZ	CO_2_ eq	26 Mt/year (35% of transport moves from car to bicycle)	Morbidity and Mortality
Maizlish et al. [15], 2013 San Francisco, CA, USA	CO_2_, PM_2.5_	4.04 million tons/year (14% reduction in transportation emissions)	DALYs
Michaelowa & Dransfeld, [11], 2008 OECD	CO_2_	100 Mt/year (N/A)	Weight Gain
Rabl & de Nazelle, [16], 2012 Europe	CO_2_ eq	1 tonnes/person/year (N/A)	Mortality
Rojas-Rueda et al. [17], 2012 Spain	CO_2_, PM_2.5_	203,251 tons/year (40% fewer car trips)	All-cause Mortality
Woodcock et al. [18], 2009 UK & India	CO_2_, PM_2.5_	0.35–0.48 tonnes/person/year (46.7–56% reduction in transport emissions)	DALYs
Woodcock et al. [15], 2013 UK	CO_2_	16 Mt–50 Mt/person (26–73% reduction in transport emissions)	Morbidity and Mortality
Lifestyle-Related Mitigation Strategy—DIET			
Aston et al. [19], 2012 UK	CO_2_ eq	27.8 Mt/year (3% current country total)	DALYs
Berners-Lee et al. [20], 2012 UK	CO_2_ eq	40 Mt/year (22–26% reduction in diet-related emissions)	Caloric and Nutrient Requirements
Biesbroek et al. [21], 2014 Dutch	CO_2_ eq	155.4 kg/person/year (4–12% of diet-related emissions)	Mortality
Briggs et al. [22], 2013 UK	CO_2_ eq	18.683 Mt/year (40% reduction of agricultural emissions)	Mortality
de Carvalho et al. [23], 2013 Brazil	CO_2_ eq	9.035 Mt/year (50% of meat production-related emissions)	Caloric and Nutrient Requirements
Edjabou & Smed, [24], 2013 Denmark	CO_2_ eq	112 kg/person/year (4–7.9%)	Caloric and Nutrient Requirements
Friel et al. [25], 2009 UK/Brazil	CO_2_ eq	9 Mt/year (30% of livestock production emissions)	DALYs
González et al. [26], 2011 Sweden	CO_2_ eq	N/A	Protein Consumption
Hallström et al. [27], 2014 Sweden	CO_2_ eq	0.2–0.4 tonnes/person/year (33–66% reduction in animal production emissions)	Caloric and Nutrient Requirements
Hendrie et al. [28], 2014 Australia	CO_2_ eq	3.6 kg/person/day (24.8% reduction in diet-related emissions)	Caloric and Nutrient Requirements
Hoolohan et al. [29], 2013 UK	CO_2_ eq	2.2 kg/person/day (25% reduction in diet-related emissions)	Caloric and Nutrient Requirements
Macdiarmid et al. [30], 2012 UK	CO_2_ eq	1.37 kg/person/day (36% reduction in diet-related emissions)	Caloric and Nutrient Requirements
Masset et al. [31], 2014 France	GHGE	N/A	Caloric and Nutrient Requirements
Michaelowa & Dransfeld, [11], 2008 OECD	CO_2_ eq	20 Mt/year (N/A)	Nutrient (fat) Consumption
Pairotti et al. [32], 2015 Italy	CO_2_ eq	27.46 kg/family/year (6.81% reduction at the family level)	Caloric and Nutrient Requirements (values not reported)
Saxe et al. [33], 2013 Denmark	CO_2_ eq	130 kg/person/year (8% reduction in diet-related emissions)	Caloric and Protein Requirements
Scarborough et al. [34], 2012 UK	GHGE	1.7–10.9 Mt/year * (3–19% reduction in UK agricultural emissions)	Mortality
Tukker et al. [35], 2011 Europe	Environmental Impact	4876 Mt/year (25–27% reduction in diet-related impacts)	Nutrient Requirements
van Dooren et al. [36], 2014 Netherlands	CO_2_ eq	0.5 kg/person/day (11% reduction in diet-related emissions)	Nutrient Requirements
Vieux et al. [37], 2012 France	CO_2_ eq	3789 g/day/person (7.2% reduction in diet-related emissions)	Caloric Requirements
Wallén et al. [38], 2004 Sweden	CO_2_ eq	855 kg/person/year (5.4% reduction in diet-related emissions)	Caloric and Nutrient Requirements
Westhoek et al. [39], 2014 EU	GHGE	196 Mt/year (42% reduction in diet-related emissions)	Caloric and Nutrient Requirements
Wilson et al. [40], 2013 NZ	CO_2_ eq	8.48 kg/day/person (84% reduction in diet-related emissions)	Caloric and Nutrient Requirements

DALYs = Disability Adjusted Life Years; CO_2_ = Carbon Dioxide; CO_2_ eq = Carbon Dioxide Equivalents; PM_2.5_ = Particulate Matter ≤ 2.5 µm in aerodynamic diameter; GHG = Greenhouse Gas; Environmental Impact is a score based on abiotic depletion, global warming, ozone layer depletion, human toxicity, ecotoxicity, photochemical oxidation, acidification, and eutrophication; * Derived from 57.3 Mt/year agriculture emissions reported for 2005 in the 2013 UK Green House Gas Emissions Final Figur https://www.gov.uk/government/uploads/system/uploads/attachment_data/file/407432/20150203_2013_Final_Emissions_statistics.pdf.

**Table 3:** Active Transport Articles—Mitigation Strategies and Health Determinants.

Article [Reference], Year	GHGE Mitigation Strategies			Health Determinants Measured		
	LE	AT	PT	Phys Activity	Air Pollution	Traffic Injury
Grabow et al. [12], 2012	−	+	−	HEAT model for all-cause mortality from biking	Modeled PM_2.5_ and ozone levels associated with mortality, disease, hospital admissions, work-loss, and school-loss days	Not included
Lindsay et al. [13], 2011	−	+	−	HEAT all-cause mortality	HAPiNZ morbidity and mortality from PM_10_, NO_2_, and CO	mortality
Macmillan et al. [14], 2014	+	+	−	All-cause mortality from increased biking	HAPiNZ estimates for death, disease, reduced activity, and hospitalization based on PM_10_, CO, and benzene	All-cause mortality from increased biking
Maizlish et al. [15], 2013	+	+	−	Relative risk of disease based on METs, reported in YLL and YLD	Relative risk of disease based on PM_2.5_ levels	YLL and YLD
Michaelowa & Dransfeld, [11], 2008	−	+	−	Obesity prevention estimates	Not included	Not included
Rabl & de Nazelle, [16], 2012	−	+	−	HEAT model for all-cause mortality for biking and extended to walking, monetized	ExternE for air pollution mortality, monetized	Mortality based on statistics from Paris, Belgium, and the Netherlands, monetized
Rojas-Rueda et al. [17], 2012	−	+	+	HEAT model for all-cause mortality	Relative risk of all-cause mortality based on PM_2.5_ levels	Relative risk of all-cause mortality
Woodcock et al. [18], 2009	+	+	−	Relative risk of disease based on METs	Relative risk of disease based on PM_2.5_ levels	Total number of accidents, reported in YLL and YLD
Woodcock et al. [15], 2013	+	+	+	Relative risk of disease based on METs, reported in YLL and YLD	Relative risk of disease based on PM_2.5_ levels	Total number of accidents, reported in YLL and YLD

AT—Active Transport; CO = Carbon Monoxide; ExternE = External Cost of Energy; HAPiNZ = Health and Air Pollution New Zealand; HEAT = Health Economic Assessment Tool; LE—Low Carbon Emissions Vehicles; METs = metabolic equivalent of task; NO_2_ = Nitrogen dioxide; PM_2.5_ = Particulate Matter ≤ 2.5 µm in aerodynamic diameter; PM_10_ = Particulate Matter ≤ 10 µm in aerodynamic diameter; PT—Public Transport; YLD = years lost due to disability; YLL = years of life lost.

**Table 4:** Diet Articles—Meat reduction and substitution methods.

Article [Reference], Year	Meat Reduction	Substitution
Aston et al. [19], 2012	Double vegetarians, remaining consume meat at level of the bottom quintile	Energy adjusted intake for population energy demands
Berners-Lee et al. [20], 2012	Vegan and vegetarian diets adopted by all	Per capita energy consumption is maintained equivalent to current
Biesbroek et al. [21], 2014	Meat consumption reduced by 1/3	1/3 meat calories substituted with other foods
Briggs et al. [22], 2013	Taxing high GHG emitting food groups, results in reduced meat consumption	Substituted with low GHG emitting food groups (DIETRON model)
De Carvalho et al. [23], 2013	Reduction of meat consumption to recommended levels according to the World Cancer Research Fund (71.4 g/day)	No
Edjabou & Smed, [24], 2013	Taxing high GHG emitting food groups, results in reduced meat consumption	Substituted with low GHG emitting food groups
Friel et al. [25], 2009	30% reduction in meat production in the UK was assumed to result in reduced consumption	No
González et al. [26], 2011	Vegetarianism is promoted due to efficiency (low GHG emissions) per gram of protein delivered relative to meat	Protein delivery via legumes and other vegetable sources is considered
Hallström et al. [27], 2014	Meat is reduced to recommended daily intake (RDI), about 25% reduction across the population	No
Hendrie et al. [28], 2014	Reduced meat and elimination of non-core foods were considered	Yes, alternative diets were considered in their entirety and compared to RDI
Hoolohan et al. [29], 2013	Meat consumption is reduced in 5% increments	Substitution was spread across “realistic plant-based” alternatives
Macdiarmid et al. [30], 2012	Linear programming model selected realistic diet that minimized GHG emission, partially via reduction in meat consumption	Yes, “realistic” scenarios to meet nutritional needs while minimizing GHG emissions were designed
Masset et al. [31], 2014	Reduction in meat consumption is advised based on price and GHG emissions	Yes, alternative foods with low cost and GHG emissions are identified, although exact substitution is not enumerated
Michaelowa and Dransfeld, [11], 2008	Observed reduction in beef consumption from 1990 to 2005 is thought to have resulted in decreased GHG emission	No
Pairotti et al. [32], 2015	Meat consumption was reduced in all alternative diet scenarios	Consumption of substitute foods was considered, but not always with the same energy provision
Saxe et al. [33], 2013	All diet scenarios included a reduction in meat consumption	Substitutions were made such that all diets had equal energy and protein content
Scarborough et al. [34], 2012	All diet scenarios include reductions in meat consumption	All diet scenarios include replacement with lower GHG emitting meats or other foods
Tukker et al. [35], 2011	Recommended diet and reduced meat diets were considered.	Yes, substitution with protein and energy consumption constant across all scenarios
Van Dooren et al. [36], 2014	All diets include reduced meat. Semi- and pesco-vegetarian diets are recommended for health and GHG emissions.	Yes, substitution is included to maintain the same level of energy in each diet.
Vieux et al. [37], 2012	Meat reduction scenarios: consumption reduced by 20% for those eating >50 g/day or reduction to a maximum of 50 g/day	Meat substitution was accounted for in some scenarios by general increase across other food categories.
Wallén et al. [38], 2004	The sustainable diet suggests a 36% reduction in average meat consumption.	Yes, more sustainable foods are increased to compensate for decreased meat consumption.
Westhoek et al. [39], 2014	Scenarios model as 25–50% reduction in animal-derived foods.	Yes, substitution with plant-based foods were made to provide equivalent energy
Wilson et al. [40], 2013	Linear programming was used to select diets low in cost and emissions, meat was eliminated on the basis of health and GHG emissions unless specifically required in the diet scenario.	Meat was replaced by healthier and lower emissions foods when meat requirements were removed.

**Table 5:** Biases, limitations, and uncertainties rubric scores by lifestyle-related mitigation category.

Rubric Category	Active Transport (AT)	Diet	AT and Diet
General (5/5) = 100%	4.22/5 or 84.4%	3.55/5 or 71.1%	3.77/5 or 75.3%
Emissions (5/5) = 100%	3.66/5 or 73.3%	4.19/5 or 83.9%	4.05/5 or 80.9%
Health (10/10) = 100%	7.11/10 or 71.1%	4.56/10 or 45.6%	5.28/10 or 52.8%
Total (20/20) = 100%	15/20 or 75.0%	12.34/20 or 61.7%	13.1/20 or 65.5%

**Table 6:** Average health rubric scores by health outcome measure.

Health Outcome Measure	Average Health Score	Health Score Range
Calories (*n* = 15)	4/10	1–7
Nutrient (*n* = 14)	3.8/10	1–7
Morbidity (*n* = 5)	7.2/10	7–8
Mortality (*n* = 12)	7.5/10	3–10

## Supplementary Materials

The following are available online at www.mdpi.com/1660-4601/14/5/468/s1, Table S1: Detailed summary of reviewed articles and their measured health and GHGE outcomes; Table S2: Types of dietary changes examined in reviewed articles; Table S3: A summary of health outcomes evaluated in each article; Table S4: A summary of the modeling tools used to estimate physical activity impact on health in reviewed articles.

## Abbreviations

GHGE	Greenhouse Gas Emissions
IPCC	Intergovernmental Panel on Climate Change
CO_2_	carbon dioxide
METs	Metabolic Equivalent of Task
DALYs	Disability-Adjusted Life Years
LCAs	Lifecycle Assessments
OECD	Organization for Economic Co-operation and Development

## References

[1] An Action Agenda for Sustainable Development. http://unsdsn.org/resources/goals-and-targets/.

[2] Field C.B., Barros V.R., Dokken D.J., Mach K.J., Mastrandrea M.D., Bilir T.E., Chatterjee M., Ebi K.L., Estrada Y.O., Genova R.C., IPCC (2014). Summary for policymakers. Climate Change 2014: Impacts, Adaptation, and Vulnerability. Part A: Global and Sectoral Aspects. Contribution of Working Group II to the Fifth Assessment Report of the Intergovernmental Panel on Climate Change.

[3] Sources of Greenhouse Gas Emissions. http://www.epa.gov/climatechange/ghgemissions/sources.html.

[4] Pichs-Madruga O.R., Sokona Y., Farahani E., Kadner S., Seyboth K., Adler A., Baum I., Brunner S., Eickemeier P., Kriemann B., IPCC (2014). Summary for Policymakers. Climate Change 2014, Mitigation of Climate Change. Contribution of Working Group III to the Fifth Assessment Report of the Intergovernmental Panel on Climate Change.

[5] Whitmarsh L., Seyfang G., O’Neill S. (2011). Public engagement with carbon and climate change: To what extent is the public “carbon capable”?. Glob. Environ. Chang..

[6] Hallal P.C., Andersen L.B., Bull F.C., Guthold R., Haskell W., Ekelund U. (2012). Global physical activity levels: Surveillance progress, pitfalls, and prospects. Lancet.

[7] Lock K., Pomerleau J., Causer L., Altmann D.R., McKee M. (2005). The global burden of disease attributable to low consumption of fruit and vegetables: Implications for the global strategy on diet. Bull. World Health Organ..

[8] World Health Organization (WHO) (2009). Global Health Risks: Mortality and Burden of Disease Attributable to Selected Major Risks.

[9] Yip C.S.C., Crane G., Karnon J. (2013). Systematic review of reducing population meat consumption to reduce greenhouse gas emissions and obtain health benefits: Effectiveness and models assessments. Int. J. Public Health.

[10] Xia T., Zhang Y., Crabb S., Shah P. (2013). Cobenefits of replacing car trips with alternative transportation: A review of evidence and methodological issues. J. Environ. Public Health.

[11] Michaelowa A., Dransfeld B. (2008). Greenhouse gas benefits of fighting obesity. Ecol. Econ..

[12] Grabow M.L., Spak S.N., Holloway T., Stone B., Mednick A.C., Patz J.A. (2012). Air quality and exercise-related health benefits from reduced car travel in the midwestern United States. Environ. Health Perspect..

[13] Lindsay G., Macmillan A., Woodward A. (2011). Moving urban trips from cars to bicycles: Impact on health and emissions. Aust. N. Z. J. Public Health.

[14] Macmillan A., Connor J., Witten K., Kearns R., Rees D., Woodward A. (2014). The societal costs and benefits of commuter bicycling: Simulating the effects of specific policies using system dynamics modeling. Environ. Health Perspect..

[15] Maizlish N., Woodcock J., Co S., Ostro B., Fanai A., Fairley D. (2013). Health cobenefits and transportation-related reductions in greenhouse gas emissions in the San Francisco Bay area. Am. J. Public Health.

[16] Rabl A., De Nazelle A. (2012). Benefits of shift from car to active transport. Transp. Policy.

[17] Rojas-Rueda D., De Nazelle A., Teixidó O., Nieuwenhuijsen M. (2012). Replacing car trips by increasing bike and public transport in the greater Barcelona metropolitan area: A health impact assessment study. Environ. Int..

[18] Woodcock J., Edwards P., Tonne C., Armstrong B.G., Ashiru O., Banister D., Beevers S., Chalabi Z., Chowdhury Z., Cohen A. (2009). Public health benefits of strategies to reduce greenhouse-gas emissions: Urban land transport. Lancet.

[19] Aston L.M., Smith J.N., Powles J.W. (2012). Impact of a reduced red and processed meat dietary pattern on disease risks and greenhouse gas emissions in the UK: A modelling study. BMJ Open.

[20] Berners-Lee M., Hoolohan C., Cammack H., Hewitt C. (2012). The relative greenhouse gas impacts of realistic dietary choices. Energy Policy.

[21] Biesbroek S., Bueno-de-Mesquita H.B., Peeters P.H., Verschuren W.M., van der Schouw Y.T., Kramer G.F., Tyszler M., Temme E.H. (2014). Reducing our environmental footprint and improving our health: Greenhouse gas emission and land use of usual diet and mortality in EPIC-NL: A prospective cohort study. Environ. Health.

[22] Briggs A.D., Kehlbacher A., Tiffin R., Garnett T., Rayner M., Scarborough P. (2013). Assessing the impact on chronic disease of incorporating the societal cost of greenhouse gases into the price of food: An econometric and comparative risk assessment modelling study. BMJ Open.

[23] De Carvalho A.M., Cesar C.L., Fisberg R.M., Marchioni D.M. (2013). Excessive meat consumption in Brazil: Diet quality and environmental impacts. Public Health Nutr..

[24] Edjabou L.D., Smed S. (2013). The effect of using consumption taxes on foods to promote climate friendly diets–The case of Denmark. Food Policy.

[25] Friel S., Dangour A.D., Garnett T., Lock K., Chalabi Z., Roberts I., Butler A., Butler C.D., Waage J., McMichael A.J. (2009). Public health benefits of strategies to reduce greenhouse-gas emissions: Food and agriculture. Lancet.

[26] González A.D., Frostell B., Carlsson-Kanyama A. (2011). Protein efficiency per unit energy and per unit greenhouse gas emissions: Potential contribution of diet choices to climate change mitigation. Food Policy.

[27] Hallström E., Röös E., Börjesson P. (2014). Sustainable meat consumption: A quantitative analysis of nutritional intake, greenhouse gas emissions and land use from a Swedish perspective. Food Policy.

[28] Hendrie G.A., Ridoutt B.G., Wiedmann T.O., Noakes M. (2014). Greenhouse Gas Emissions and the Australian Diet—Comparing Dietary Recommendations with Average Intakes. Nutrients.

[29] Hoolohan C., Berners-Lee M., McKinstry-West J., Hewitt C. (2013). Mitigating the greenhouse gas emissions embodied in food through realistic consumer choices. Energy Policy.

[30] Macdiarmid J.I., Kyle J., Horgan G.W., Loe J., Fyfe C., Johnstone A., McNeill G. (2012). Sustainable diets for the future: Can we contribute to reducing greenhouse gas emissions by eating a healthy diet?. Am. J. Clin. Nutr..

[31] Masset G., Soler L.-G., Vieux F., Darmon N. (2014). Identifying sustainable foods: The relationship between environmental impact, nutritional quality, and prices of foods representative of the French diet. J. Acad. Nutr. Diet..

[32] Pairotti M.B., Cerutti A.K., Martini F., Vesce E., Padovan D., Beltramo R. (2015). Energy consumption and GHG emission of the Mediterranean diet: A systemic assessment using a hybrid LCA-IO method. J. Clean. Prod..

[33] Saxe H., Larsen T.M., Mogensen L. (2013). The global warming potential of two healthy nordic diets compared with the average danish diet. Clim. Chang..

[34] Scarborough P., Allender S., Clarke D., Wickramasinghe K., Rayner M. (2012). Modelling the health impact of environmentally sustainable dietary scenarios in the UK. Eur. J. Clin. Nutr..

[35] Tukker A., Goldbohm R.A., De Koning A., Verheijden M., Kleijn R., Wolf O., Pérez-Domínguez I., Rueda-Cantuche J.M. (2011). Environmental impacts of changes to healthier diets in Europe. Ecol. Econ..

[36] Van Dooren C., Marinussen M., Blonk H., Aiking H., Vellinga P. (2014). Exploring dietary guidelines based on ecological and nutritional values: A comparison of six dietary patterns. Food Policy.

[37] Vieux F., Darmon N., Touazi D., Soler L.G. (2012). Greenhouse gas emissions of self-selected individual diets in France: Changing the diet structure or consuming less?. Ecol. Econ..

[38] Wallén A., Brandt N., Wennersten R. (2004). Does the swedish consumer’s choice of food influence greenhouse gas emissions?. Environ. Sci. Policy.

[39] Westhoek H., Lesschen J.P., Rood T., Wagner S., De Marco A., Murphy-Bokern D., Leip A., van Grinsven H., Sutton M.A., Oenema O. (2014). Food choices, health and environment: Effects of cutting Europe’s meat and dairy intake. Glob. Environ. Chang..

[40] Wilson N., Nghiem N., Mhurchu C.N., Eyles H., Baker M.G., Blakely T. (2013). Foods and dietary patterns that are healthy, low-cost, and environmentally sustainable: A case study of optimization modeling for New Zealand. PLoS ONE.

[41] Woodcock J., Givoni M., Morgan A.S. (2013). Health impact modelling of active travel visions for England and Wales using an Integrated Transport and Health Impact Modelling tool (ITHIM). PLoS ONE.

[42] Andersen Z.J., de Nazelle A., Mendez M.A., Garcia-Aymerich J., Hertel O., Tjønneland A., Overvad K., Raaschou-Nielsen O., Nieuwenhuijsen M.J. (2015). A Study of the Combined Effects of Physical Activity and Air Pollution on Mortality in Elderly Urban Residents: The Danish Diet, Cancer, and Health Cohort. Environ. Health Perspect..

[43] Food and Agriculture Organization of the United Nations-FAO (2010). Sustainable Diets and Biodiversity: Directions and Solutions for Policy, Research and Action. Proceedings of the International Scientific Symposium: Biodiversity and Sustainable Diets United Against Hunger.

[44] Remais J.V., Hess J.J., Ebi K.L., Markandya A., Balbus J.M., Wilkinson P., Haines A., Chalabi Z. (2014). Estimating the health effects of greenhouse gas mitigation strategies: Addressing parametric, model, and valuation challenges. Environ. Health Perspect..

[45] Tucker P., Gilliland J. (2007). The effect of season and weather on physical activity: A systematic review. Public Health.

[46] Wanner M., Götschi T., Martin-Diener E., Kahlmeier S., Martin B.W. (2012). Active transport, physical activity, and body weight in adults: A systematic review. Am. J. Prev. Med..

[47] Cohen A.J., Anderson H.R., Ostro B., Dev Pandey K., Krzyzanowski M., Künzli N., Gutschmidt K., Pope C.A., Romieu I., Samet J.M., Ezzati M., Lopez A.D., Rodgers A., Murray C.J.L. (2004). Urban air pollution. Comparative Quantification of Health Risks: Global and Regional Burden of Disease Attributable to Selected Major Risk Factors.

[48] McMichael A.J., Powles J.W., Butler C.D., Uauy R. (2007). Food, livestock production, energy, climate change, and health. Lancet.

