# Supplementary material for: Assessing Greenhouse Gas Emissions and Health Co-Benefits: A Structured Review of Lifestyle-Related Climate Change Mitigation Strategies
Source: Int J Environ Res Public Health. 2017 Apr 27;14(5):468. doi: 10.3390/ijerph14050468 (PMC5451919; doi:10.3390/ijerph14050468)
Supplement: Supplementary file 1 [file ijerph-14-00468-s001.pdf]

Assessing Greenhouse Gas Emissions and Health Co-Benefits: A Structured Review of Lifestyle-Related Climate Change Mitigation Strategies

Vivian G.M. Quam, Joacim Rocklöv, Mikkel B.M. Quam and Rebekah A. I. Lucas

Table S1. Summary of reviewed articles and their measured outcomes.

Article	Geography	Modeled or observational data	Health outcome	GHG/emission outcome
Lifestyle-Related Mitigation Strategy – ACTIVE TRANSPORT				
Grabow *et al.* 2012	Midwest US (urban and suburban)	Modeled elimination of short car trips, replacing 50% of trips <= 8Km by bike	BenMAP estimated health impact due to CMAQ-simulated changes in ambient air pollution. Monetized all-cause mortality using HEAT	PM and Ozone using CMAQ model. Modeled changes in primary emissions (NOx, CO, sulfur dioxide, ammonia, VOCs, elemental and organic carbon, fine and course PM) as well as emissions from evaporation, brake dust, re-suspended road dust. And refueling
Lindsay *et al.* 2011	New Zealand	Modeled scenarios where bike trips replaced short car trips (<7km) at different rates	Mortality from PA, air pollution, and traffic accidents. Monetized.	CO_2 eq emissions
Macmillan *et al.* 2014	Auckland, New Zealand	Modeled scenarios for injury, physical activity, fuel costs, air pollution, and carbon emissions	For adults > 30: death, cardio and resp hospitalization due to PM and CO. Monetized all-cause mortality due to cycling change.	Monetized carbon emissions in CO_2 eq, including CO_2, CO, CH4, and NOx
Maizlish *et al.* 2013	San Francisco Bay Area, CA	Modeled scenarios for travel mode shift from car to AT	DALYS due to physical activity, air pollution and traffic accidents. DALYs due to cardio disease, diabetes, dementia, breast cancer, colon cancer, and depression	Greenhouse Gas Emissions
Michaelowa & Dransfeld 2008	Global	Models shift of 2.5 km /person /day from motorized to	Reduced obesity	CO_2 eq emissions

		non-motorized transport		
Rabl & de Nazelle 2012	Europe	Modeled scenarios where car travel to work is replaced by walking for biking	Mortality from PA, traffic accidents, and pollution. Monetized	CO_2 eq emissions monetized
Rojas-Rueda *et al.* 2012	Barcelona, Spain	Modeled 8 scenarios with varying levels of car, bike and public transport	All-cause mortality and change in life expectancy due to PA, air pollution, and traffic. As well as PM for the general population.	CO_2
Woodcock *et al.* 2009	London, UK and Delhi, India	Modeled scenarios with AT and low emission vehicles and a combination of the two. 4 scenarios compared to BAU.	Comparative risk assessment. DALYS due to PA, air pollution (PM 2.5µm or less) and traffic accidents were measured.	CO_2 emissions from motor vehicle fuel combustion. No LCA.
Woodcock *et al.* 2013	England and Wales	Integrated transport and health impact modeling tool	DALYS for physical activity, air pollution, and traffic accidents using HEAT	CO_2 emissions
Lifestyle-Related Mitigation Strategy – DIET				
Aston *et al.* 2012	UK	Modeled conversion of "high meat" diets to "low meat diets"	Reduction in risk of coronary heart disease, diabetes, and colorectal cancer	GHG emissions converted to CO_2 eq
Berners-Lee *et al.* 2012	UK	Modeled 6 vegetarian/ vegan diets compared to BAU	Consumption of protein, carbohydrates, added sugar, fat, sodium	GHG emissions converted to CO_2 eq
Biesbroek *et al.* 2014	Netherlands	Estimated GHGE and land use from food frequency questionnaire	Mortality	CO_2 eq
Briggs *et al.* 2013	UK	Modeled tax scenarios where food and drink with above average GHG emission are taxed with or without subsidizing those with below average	Deaths averted	Emissions in CO_2 eq
de Carvalho *et al.* 2013	Brazil	Estimated GHGE associated with red meat consumption	Consumption of red meat, diet quality	CO_2 eq emissions
Edjabou & Smed 2013	Denmark	Modeled tax scenarios	Nutrient consumption including energy, saturated fat, and added sugar	CO_2 eq emissions
Friel *et al.* 2009	UK and Brazil	Modeled scenarios to achieve 50%	DALYS from ischemic heart disease and stroke	CO_2 eq

		reduction in emissions in the food and agriculture sector with technology and consumption changes		
González *et al.* 2011	Sweden	Observational data	G Protein per kg food eaten	CO_2 eq and energy inputs per kg food
Hallström *et al.* 2014	Sweden	2 Modeled dietary scenarios in line with RDI	Dietary nutrients consumed	CO_2 eq emissions and land requirement
Hendrie *et al.* 2014	Australia	Modelled GHGE from average Australian diet and compared to recommended diets	Cost, nutritional benefit, calories, non-core foods	CO_2 eq emissions
Hoolohan *et al.* 2013	UK	Modeling reduction of waste, elimination of meat, shift to less intensive meat, or avoiding hot-house and air-freighted foods	Nutrient intake	CO_2 eq emissions
Macdiarmid *et al.* 2012	UK	Database created linking nutrient composition and GHGE data for 82 food groups	Nutrient composition	CO_2 eq
Masset *et al.* 2014	France	Correlations between nutritional value and environmental impact were calculated	SAIN:LIM score nutrient content/recommendation ratio for protein, fiber, calcium, Vit C, and iron against sat fatty acids, added sugar, sodium	GHG emissions, acidification, and eutrophication
Michaelowa & Dransfeld 2008	Global	Models shift of 2.5 km /person /day from motorized to non-motorized transport	Reduced obesity	CO_2 eq emissions
Pairotti *et al.* (2015)	Italy	Modeled diet scenarios; Mediterranean, BAU, healthy, vegetarian	Energy consumption within food groups	CO_2 eq emissions
Saxe *et al.* 2013	Denmark	Compared 3 diets, 2 healthy Nordic diets against average Danish diet adjusted to contain similar energy and protein levels	Reduce obesity and chronic disease	CO_2 eq emissions
Scarborough *et al.* (2012)	UK	3 diet scenarios were modeled and	Mortality from cardiovascular disease	GHG emissions

		compared to BAU	and cancer	
Tukker *et al.* (2011)	Europe	3 diet scenarios were modeled according to dietary recommendations	Nutrient consumption	Abiotic resource depletion, climate change, ozone depletion, human toxicity, ecotoxicity, photochemical oxidant formation, terrestrial acidification, and freshwater eutrophication
Van Dooren *et al.* (2014)	Netherlands	Modeled 6 diets based on health and reduced animal protein consumption	Health score for each diet calculated on 10 indicators	CO_2 eq emissions and land use
Vieux *et al.* (2012)	France	Modeled diet scenarios reduced calorie consumption and substituting vegetables for meat	Caloric intake	CO_2 eq emissions
Wallén *et al.* (2004)	Sweden	Modeled suggested diets based on food availability	Consumption in calories	CO_2 eq emissions
Westhoek *et al.* (2014)	Europe	Modeled a halving of meat and dairy consumption	Nutrient intake	Nitrogen emissions, GHG, land use
Wilson *et al.* 2013	New Zealand	Modeled diet scenarios based on i) low-cost ii) low GHGE and low-cost iii) high vegetable intake iv) familiar meals	Nutrient value	GHG emissions

PM = Particulate Matter; CMAQ = Community Multiscale Air Quality Model; CO = Carbon Monoxide; NO_2 = oxides of nitrogen; VOCs = volatile organic compound; CO_2 eq = Carbon dioxide equivalents; CH_4 = Methane; DALYs = Disability Adjusted Life Years; $PM_{2.5}$ = Particulate Matter ≤ 2.5 μm in aerodynamic diameter; AT = Active transport; BAU = Business As Usual; LCA = Life Cycle Analysis; GHG = Greenhouse Gas; GHGE = Greenhouse Gas emission; HEAT = Health Economic Assessment Tool; RDI = Recommended Daily Intake.

Table S2. Lifestyle-Related Mitigation Strategy – Diet. Type of dietary change examined in reviewed articles.

**Author**	**Reduced Meat/Dairy**	**Other**
Lifestyle-Related Mitigation Strategy – DIET		
Aston *et al.* 2012	yes	no
Berners-Lee *et al.* 2012	yes	no
Biesbroek *et al.* 2014	yes	no
Briggs *et al.* 2013	yes	yes
de Carvalho *et al.* 2013	yes	no
Edjabou & Smed 2013	yes	yes

Friel *et al.* 2009	yes	no
González *et al.* 2011	yes	no
Hallström *et al.* 2014	yes	no
Hendrie *et al.* 2014	yes	yes
Hoolohan *et al.* 2013	yes	yes
Macdiarmid *et al.* 2012	yes	yes
Masset *et al.* 2014	yes	yes
Michaelowa & Dransfeld 2008	yes	yes
Pairotti *et al.* 2015	yes	yes
Saxe *et al.* 2013	yes	yes
Scarborough *et al.* 2012	yes	no
Tukker *et al.* 2011	yes	yes
van Dooren *et al.* 2014	yes	yes
Vieux *et al.* 2012	yes	no
Wallén *et al.* 2004	yes	no
Westhoek *et al.* 2014	yes	no
Wilson *et al.* 2013	yes	yes

Table S3. Health score by health outcome for reviewed articles.

**Title**	**Caloric**	**Nutrient**	**Morbidity**	**Mortality**	**Health score**
Lifestyle-Related Mitigation Strategies – ACTIVE TRANSPORT					
Grabow *et al.* 2012			yes	yes	7/10
Lindsay *et al.* 2011			yes	yes	7/10
Macmillan *et al.* 2014			yes	yes	7/10
Maizlish *et al.* 2013			yes	yes	8/10
Michaelowa & Dransfeld 2008	n/a				2/10
Rabl & de Nazelle 2012				yes	6/10
Rojas-Rueda *et al.* 2012				yes	8/10
Woodcock *et al.* 2009				yes	7/10
Woodcock *et al.* 2013				yes	10/10
Lifestyle-Related Mitigation Strategies - DIET					
Aston *et al.* 2012			yes		7/10
Berners-Lee *et al.* 2012	yes	yes			3/10
Biesbroek *et al.* 2014				yes	3/10
Briggs *et al.* 2013				yes	9/10
de Carvalho *et al.* 2013	yes	yes			3/10
Edjabou & Smed 2013	yes	yes			4/10
Friel *et al.* 2009				yes	10/10
González *et al.* 2011		yes (protein only)			3/10
Hallström *et al.* 2014	yes	yes			7/10
Hendrie *et al.* 2014	yes	yes			3/10
Hoolohan *et al.* 2013	yes	yes			4/10
Macdiarmid *et al.* 2012	yes	yes			6/10
Masset *et al.* 2014	yes	yes			1/10
Michaelowa & Dransfeld 2008	n/a				1/10

Pairotti *et al.* 2015	yes			2/10
Saxe *et al.* 2013	yes	yes		2/10
Scarborough *et al.* 2012			yes	8/10
Tukker *et al.* 2011		yes		4/10
van Dooren *et al.* 2014	yes	yes		6/10
Vieux *et al.* 2012	yes			7/10
Wallén *et al.* 2004	yes	yes		4/10
Westhoek *et al.* 2014	yes	yes		4/10
Wilson *et al.* 2013	yes	yes		4/10

Table S4. Tools for estimating physical activity impact on health in reviewed articles.

**Article**	**CRA**	**HEAT**	**System dynamics modeling**
Lifestyle-Related Mitigation Strategy – Active Transport			
Grabow *et al.* 2012		+	
Lindsay *et al.* 2011		+	
Macmillan *et al.* 2014			+
Maizlish *et al.* 2013	+		
Michaelowa & Dransfeld 2008			Unknown
Rabl & de Nazelle 2012		+	
Rojas-Rueda *et al.* 2012		+	
Woodcock *et al.* 2009	+		
Woodcock *et al.* 2013		+	

© 2017 by the authors; licensee MDPI, Basel, Switzerland. This article is an open access article distributed under the terms and conditions of the Creative Commons by Attribution (CC-BY) license (http://creativecommons.org/licenses/by/4.0/).
